# Supplementary material for: The epigenetic pioneer EGR2 initiates DNA demethylation in differentiating monocytes at both stable and transient binding sites
Source: Nat Commun. 2021 Mar 10;12:1556. doi: 10.1038/s41467-021-21661-y (PMC7946903; doi:10.1038/s41467-021-21661-y)
Supplement: Supplementary file 1 — Supplementary information. [file 41467_2021_21661_MOESM1_ESM.pdf]

Supplementary Information
for

The epigenetic pioneer EGR2 initiates DNA demethylation in differentiating monocytes at both stable and transient binding sites

Karina Mendes,[1,3] Sandra Schmidhofer,[1,4] Julia Minderjahn,[1,5] Dagmar Glatz,[1,6] Claudia Kiesewetter,[1,7] Johanna Raithel,[2] Julia Wimmer,[1,8] Claudia Gebhard,[2] Michael Rehli[1,2,*]

[1]Department of Internal Medicine III, University Hospital Regensburg, 93053 Regensburg, Germany
[2]Regensburg Centre for Interventional Immunology, University Hospital Regensburg, 93053 Regensburg, Germany

[*]Correspondence: michael.rehli@ukr.de

[3]present address: Universidade Católica Portuguesa, Center for Interdisciplinary Research in Health (CIIS), Institute of Health Sciences (ICS), Viseu, Portugal
[4]present address: AstraZeneca, Tinsdaler Weg 183, 22880 Wedel, Deutschland
[5]present address: Sandoz GmbH, Biochemiestraße 10, 6336 Langkampfen, Austria
[6]present address: Chromatin Structure and Cellular Senescence Research Unit, Maisonneuve-Rosemont Hospital Research Centre, Montréal, Quebec, Canada H1T 2M4
[7]present address: Labor Kneissler, Unterer Mühlweg 10, 93133 Burglengenfeld
[8]present address: Deutsches Patent- und Markenamt, 80331 München, Germany

Supplement Index:

Supplementary Figures 1-6 page 02-09
Supplementary Tables 1-10 page 10-19
Supplementary References page 20

Supplementary Figures & Legends

Supplementary Figure 1

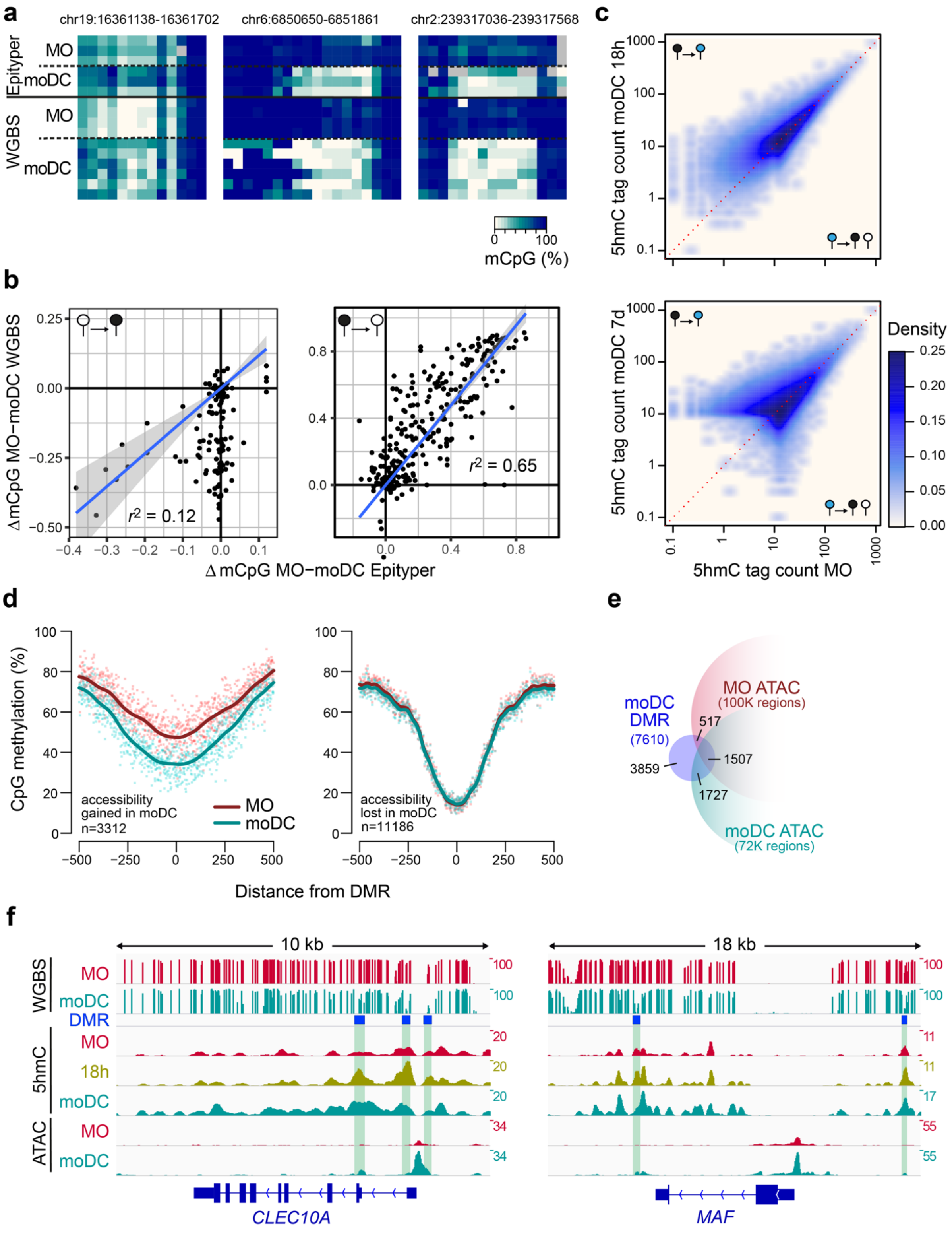

Comparative analyses of active DNA demethylation events during MO differentiation, Related to Figure 1. a Validation of whole genome bisulfite sequencing (WGBS) data of MO (n=4) and moDC (n=6) was performed using EpiTYPER DNA methylation analysis of matched MO and moDC preparations (n=3) at 38 randomly selected DMR (11 DMR methylated in moDC and 27 demethylated in moDC). Comparisons of individual methylation ratios for both technologies at three representative example regions are shown as a heatmap. Methylation ratios are indicated by coloring (white: no methylation, dark blue: 100% methylation, gray: not detected) with each column representing a single CpG. **b** Scatter plots showing correlations of methylation changes for

all individual CpGs tested between different data sets. Black lollipops indicate 5mC and white lollipops unmethylated 5C. Notably, most demethylation events were independently validated (26/27 regions tested) while only few methylation events were reproducible (2/11). **c** Scatterplots showing the density distribution of 5hmC enrichment of MO compared to moDC at 18h (top panel) or moDC at d7 (bottom panel) across a merged set of 5hmC peaks. Black lollipops indicate 5mC, blue lollipops 5hmC, and white lollipops unmethylated 5C. **d** Genomic distance distribution of averaged DNA methylation ratios centered on ATAC-seq peaks that were either lost or gained during GM-CSF/IL4-driven MO differentiation as described in Figure 1c. **e** Venn diagram showing the number of DMR demethylated in moDC overlapping ATAC-seq peaks that either lost or gained accessibility during GM-CSF/IL4-driven MO differentiation. **f** IGV genome browser tracks of example regions (*CLEC10A* and *MAF* loci) as described in Figure 1h. Highlighted DMR (green boxing) indicate regions demonstrating increased chromatin accessibility and increased 5hmC deposition during differentiation. (a,b,d) Source data are provided as a Source Data file.

Supplementary Figure 2

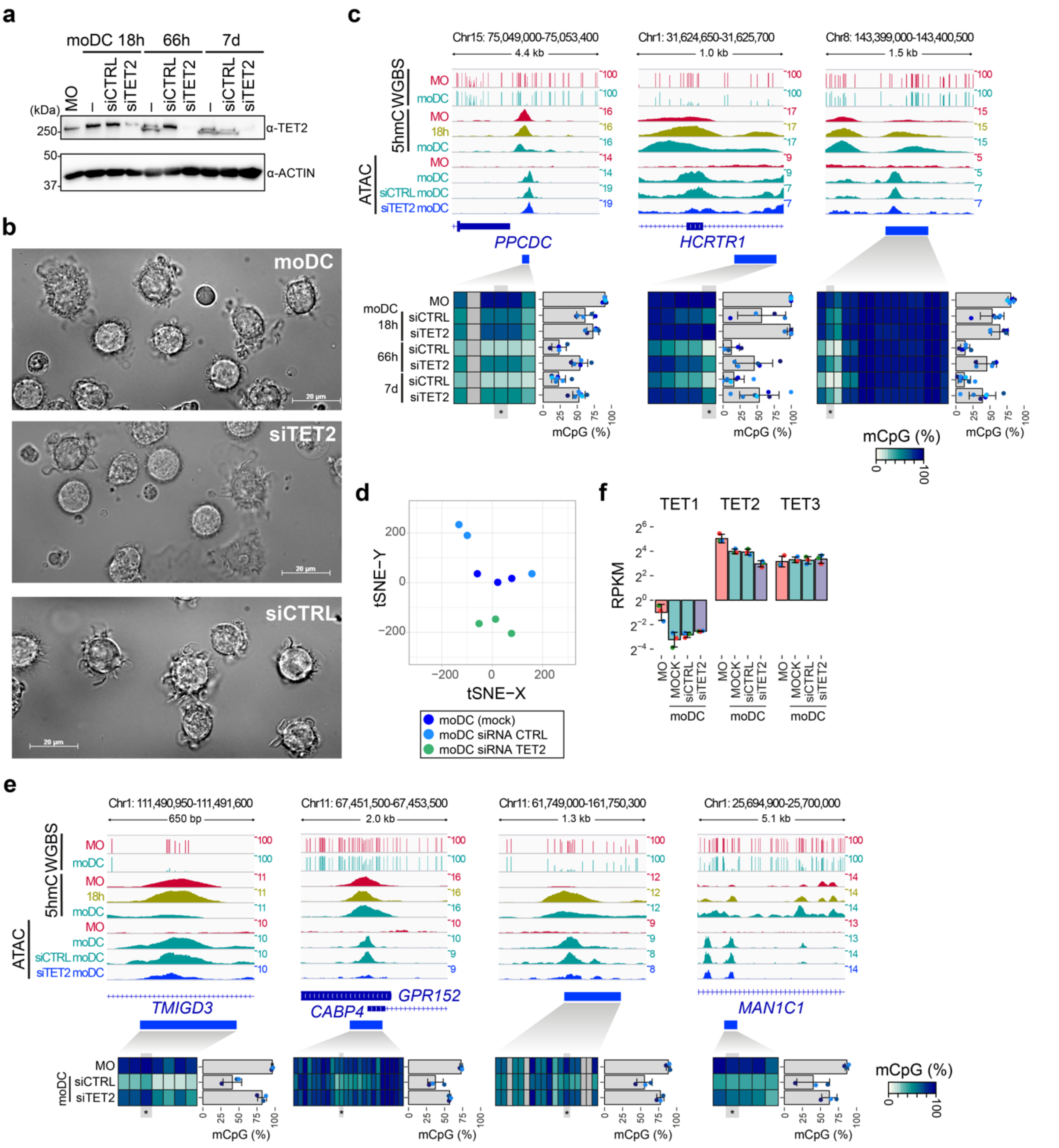

Effects of TET2 knock-down, Related to Figure 2. a TET2 protein expression levels during GM-CSF/IL4-driven MO differentiation. MO were cultured without transfection or transfected with siCTRL or siTET2 before culture and harvested at the indicated time points (18h, 66h and 7d). Blots were stained with α-TET2 and α-ACTIN antibodies (the latter represents the protein loading control). Results are representative of two independent experiments. **b** Light microscopy images of MO either untreated (moDC) or treated with siCTRL or siTET2 and cultured for 7d in the presence of GM-CSF/IL4 (60x magnification, bar represents 20 µm). Images are representative of three independent experiments. **c** IGV genome browser tracks for the indicated WGBS, 5hmC and ATAC data sets at regions around example DMRs as well as corresponding DNA methylation ratios as described in Figure 2b. Methylation ratios represent means of n=5 (18h and 66h) or n=7 (MO and 7d) biologically independent experiments ± SD. **d** Two-dimensional visualization of the RNA-seq sample distribution of the indicated treatments using tSNE embedding. Replicates of the same treatment are indicated by coloring. **e** IGV genome browser tracks for the indicated WGBS, 5hmC and ATAC data sets at regions showing reduced accessibility upon siRNA-mediated TET2 depletion. Corresponding DNA methylation ratios are shown below each genome browser track as described in Figure 2b. Methylation ratios represent means of n=3 (biologically independent experiments) ± SD. **f** Gene expression levels of TET family members in MO and MO either untreated (moDC) or treated with siCTRL or siTET2 and cultured for 7d in the presence of GM-CSF/IL4 as derived from RNA sequencing (n=3 biologically independent experiments). Reads Per Kilobase of transcript, per Million mapped reads (RPKM) were converted from normalized and batch-corrected read counts. Bars represent means ± SD, individual data points are shown as colored dots (each color represents a different donor). (a,c-f) Source data are provided as a Source Data file.

Supplementary Figure 3

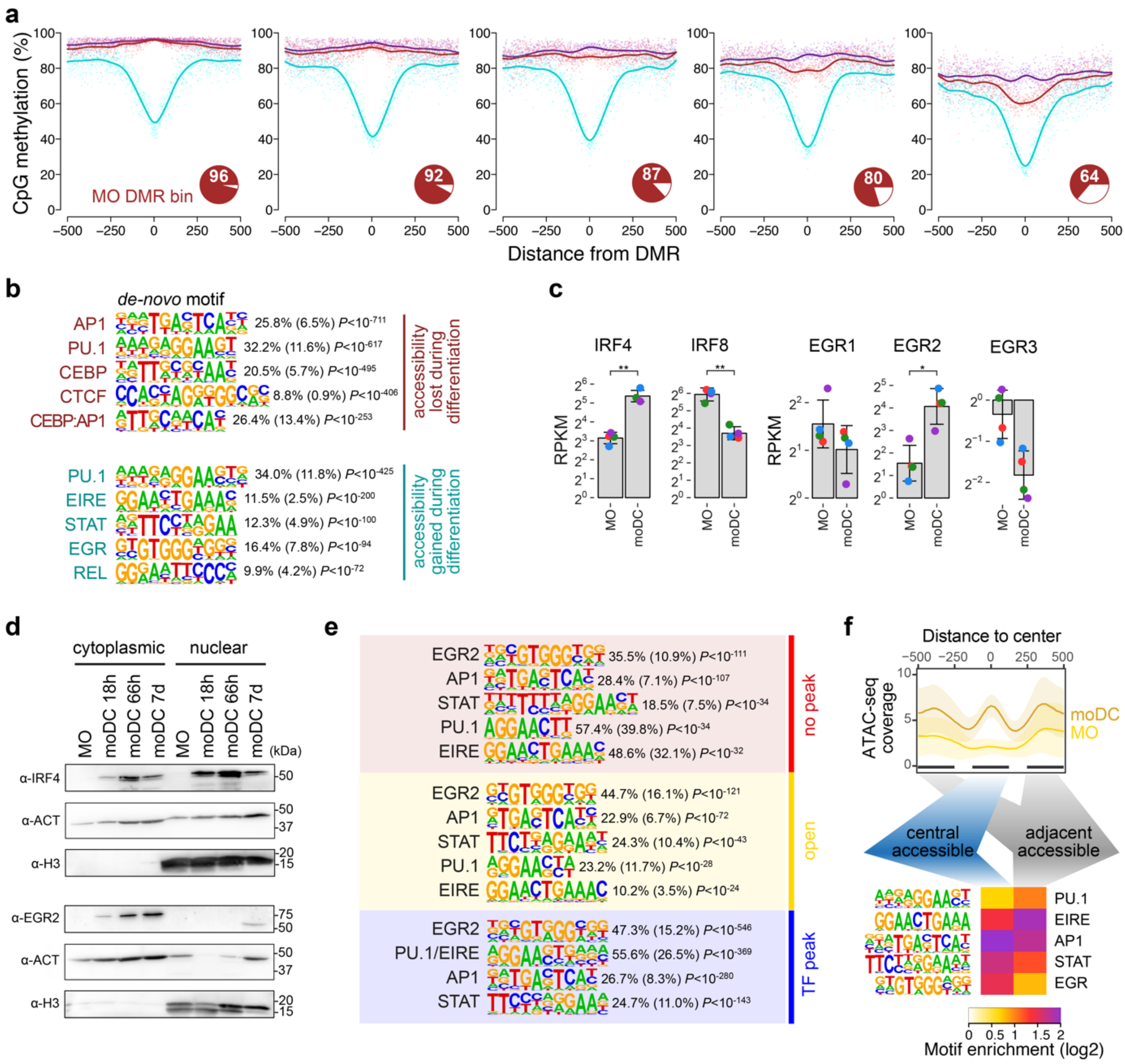

Candidate TF associated with active DNA demethylation, Related to Figure 3. a Genomic distance distributions of mean methylation ratios in HSPC (purple), MO (red) and moDC (cyan) centered on DMR demethylated in moDC. Pie charts represent the mean methylation level (in red) of DMR quantiles (1.5K each) sorted by their mean methylation ratio in MO. **b** *De novo* identified sequence motifs across MO- and moDC-specific ATAC-peaks that loose (upper panel) or gain (lower panel) chromatin accessibility during differentiation. The fraction of motifs in peaks (background values are in parenthesis) and the significance of motif enrichment (hypergeometric test) are given for the top five motifs corresponding to known motif families. **c** Gene expression levels of candidate TF family members in MO and moDC as derived from RNA sequencing (n=4 biologically independent experiments). Reads Per Kilobase of transcript, per Million mapped reads (RPKM) were converted from normalized and batch-corrected read counts. Bars represent means ± SD, individual data points are shown as colored dots (each color represents a different donor). Significance levels correspond to q-values derived from differential gene analyses using cqn and edgeR. (**q<0.01; *q<0.05; glmQLF test and BH correction for multiple testing). **d** IRF4 and EGR2 protein levels during GM-CSF/IL4-driven MO differentiation. MO were cultured for the indicated time periods (18h, 66h and 7d) and cytoplasmic and nuclear extracts were prepared and separated by SDS page. Blots were stained with the indicated antibodies (Actin in H3 represent protein loading controls). Results are representative of two independent experiments. **e** *De novo* identified sequence motifs across moDC-specific DMR in “TF peak”, “open” and “no peak” groups as defined in Figure 3c and described above in b. **f** Motif distribution in central accessible DMR and adjacent accessible regions as observed in the “open” group of moDC-specific DMR (as described in Figure 3e). Shaded bands represent the 95% confidence intervals. (a,c,d,f) Source data are provided as a Source Data file.

Supplementary Figure 4

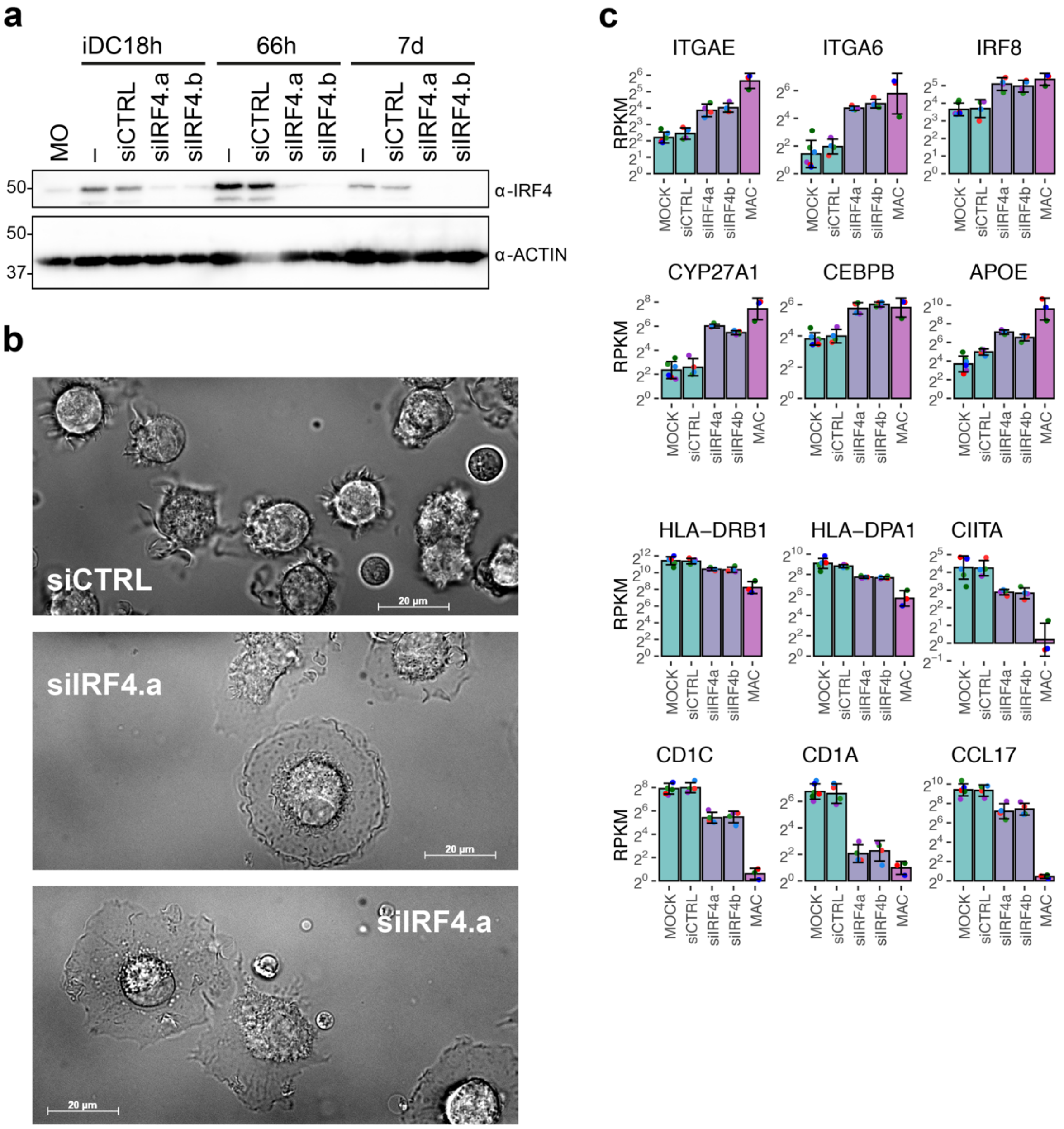

Effects of IRF4 knock-down, Related to Figure 4. a IRF4 protein expression levels during GM-CSF/IL4-driven MO differentiation. MO were cultured without transfection or transfected with siCTRL or siIRF4 (two different siRNAs) before culture and harvested at the indicated time points (18h, 66h and 7d). Blots were stained with α-IRF4 and α-ACTIN antibodies (the latter represents the protein loading control). Results are representative of two independent experiments. **b** Light microscopy images of MO treated with siCTRL or siIRF4.a and cultured for 7d in the presence of GM-CSF/IL4 (60x magnification, bar represents 20 µm). Images are representative of three independent experiments. **c** Expression levels of genes differentially regulated (FDR < 0.05) between controls and IRF4 knockdown cells. RPKM values were calculated from normalized and batch-corrected read counts of the corresponding RNA sequencing experiments. Bars represent means ± SD, individual data points are shown as colored dots (each color represents a different donor; means of n=6 (MOCK), n=4 (siCTRL, siIRF4a,b) or n=3 (MAC) biologically independent experiments). (a,c) Source data are provided as a Source Data file.

Supplementary Figure 5

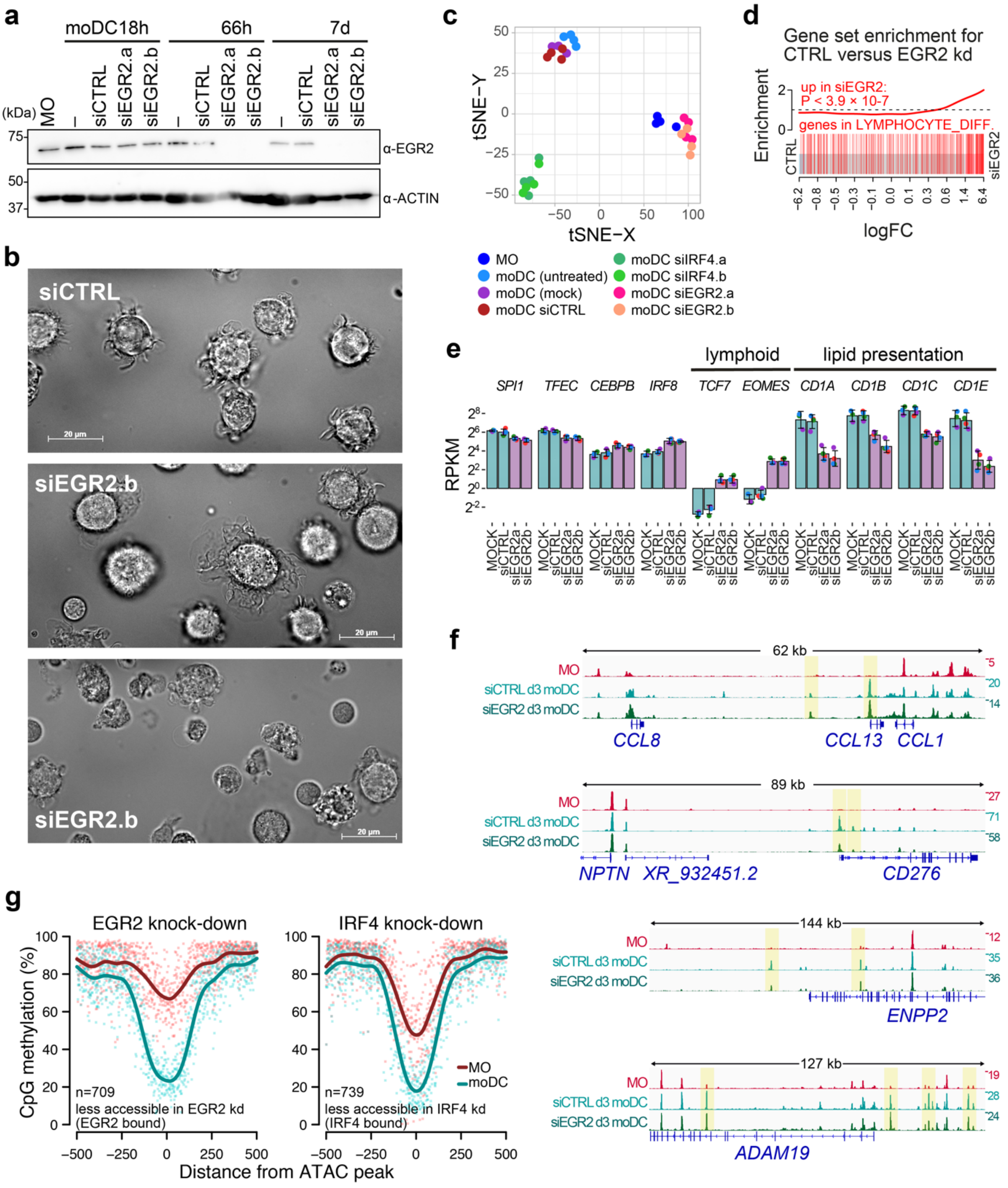

(continued on next page)

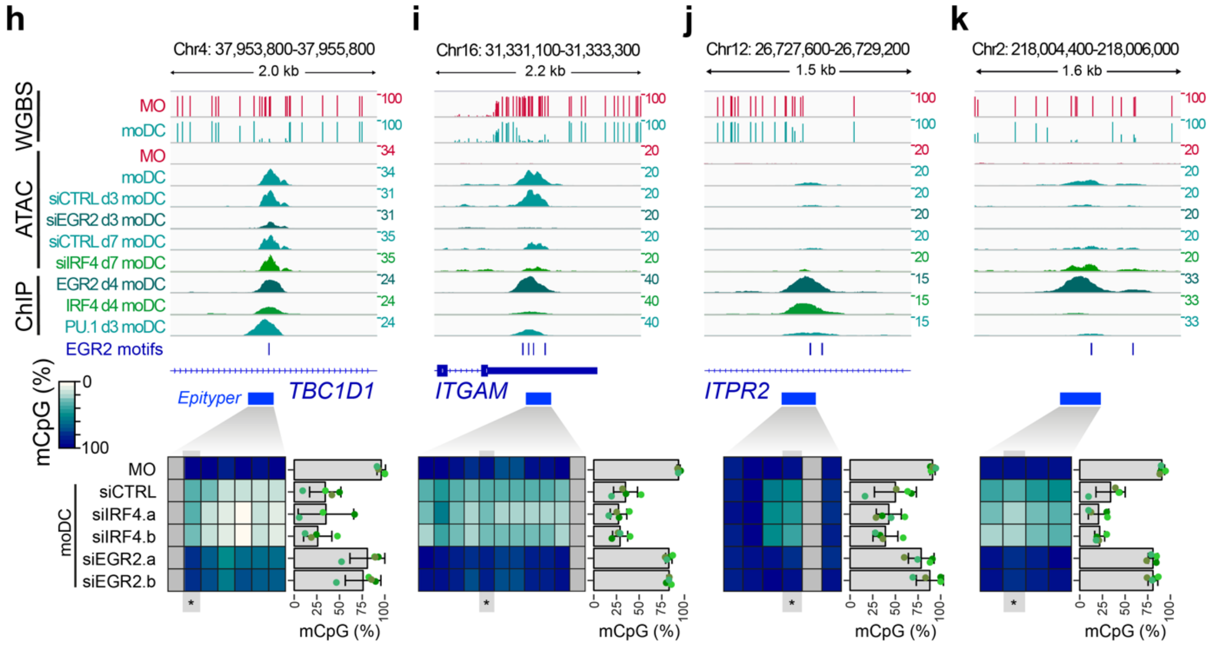

Effects of EGR2 knock-down, Related to Figure 5. a EGR2 protein expression levels in MO, moDC and siEGR2- (two different siRNAs) or siCTRL-treated moDC at the indicated time points (18h, 66h and 7d). Blots were stained with α-EGR2 and α-ACTIN antibodies (the latter represents the protein loading control). Results are representative of two independent experiments. **b** Light microscopy images of MO treated with siCTRL or siEGR2.b and cultured for 7d in the presence of GM-CSF/IL4 (60x magnification, bar represents 20 µm). Images are representative of three independent experiments. **c** Two-dimensional visualization of the RNA-seq sample distribution using tSNE embedding. Replicates of the same treatment are indicated by coloring. **d** Barcode plot presenting gene set enrichment analysis results for GO_LYMPHOCYTE_DIFFERENTIATION (MSigDB M18190, top panel) across the logFC ranked gene list of siCTRL- vs siEGR2-treated cells. **e** Comparison of expression levels of TF genes between controls and EGR2 knockdown cells. RPKM values were calculated from normalized and batch-corrected read counts of the corresponding RNA sequencing experiments. Bars represent means ± SD, individual data points are shown as colored dots (each color represents a different donor and n=3 (MOCK) or n=4 (other samples) independent experiments). **f** IGV genome browser tracks for the indicated ATAC data sets across example regions of genes that are normally induced in moDC. Chromatin accessibility gains at promoters or putative enhancers in moDC both in siCTRL- and siEGR2-treated cells are highlighted by yellow boxes. **g** Side-by-side comparison of genomic distance distributions of averaged DNA methylation ratios in MO and moDC centered on differentially accessible and TF bound sites (as introduced in Figure 5e-f). **h–k** IGV genome browser tracks for the indicated WGBS, ATAC and ChIP data sets at example regions around binding sites of EGR2, IRF4 or PU.1. Corresponding DNA methylation ratios for the indicated regions are given below. For heatmaps, methylation ratios (representing means of means of n=4 biologically independent experiments) are indicated by coloring (white: no methylation, dark blue: 100% methylation, gray: not detected) with each column representing a single CpG. For each region the data of a single CpG (highlighted and marked by asterisks) is shown. Bars represent means ± SD, individual data points are shown as colored dots (each color representing a different donor). (a,c,e,g-k) Source data are provided as a Source Data file.

Supplementary Figure 6

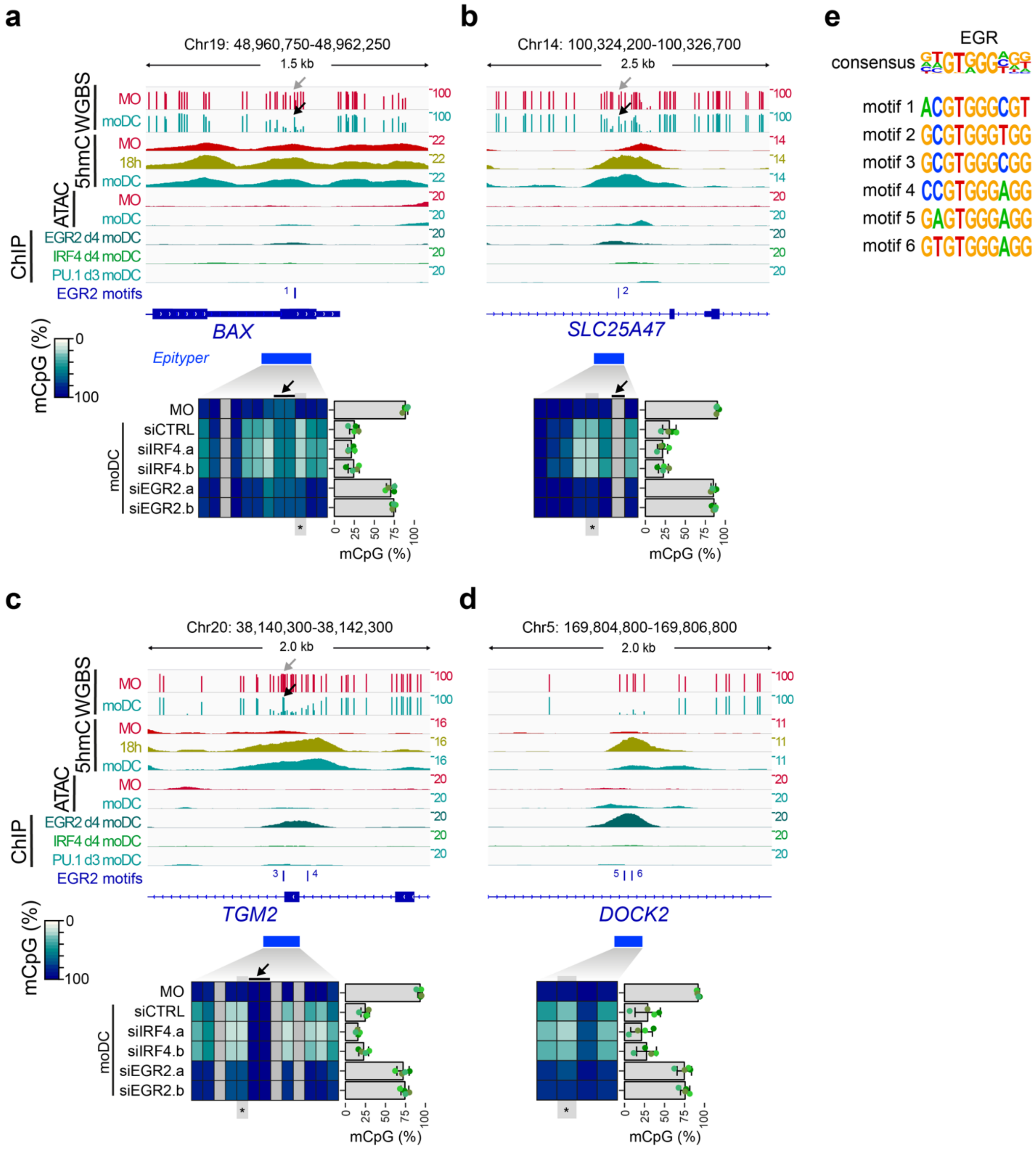

DNA methylation at largely inaccessible DMR, Related to Figure 6. a-d IGV genome browser tracks for the indicated WGBS, 5hmC, ATAC and ChIP data sets at example DMR with EGR2 consensus motifs. CpGs included in the EGR2 motif that resist demethylation are indicated above the WGBS tracks and DNA methylation heatmaps with arrows. Corresponding DNA methylation ratios (as measured by EpiTYPER) for the indicated regions are given below. For heatmaps, methylation ratios (representing means of n=4 biologically independent experiments) are indicated by coloring (white: no methylation, dark blue: 100% methylation, gray: not detected) with each column representing a single CpG. For each region the data of a single CpG (highlighted and marked by asterisks) is shown. Bars represent means ± SD, individual data points are shown as colored dots (each color representing a different donor). **e** Motif sequences for the six EGR2 motifs shown in a-d. The consensus EGR2 motif derived from DMR regions is shown on top. (a-d) Source data are provided as a Source Data file.

Supplementary Table 1
Gene blocks for cloning

Construct name	gBlock sequences (5'--3')
pEF6-3xFLAG-EGR2	**Fragment 1:**<br>TATAGGGAGACCCAAGCTGGCTAGGTAAGCTTGGTACCGAGCTCGGATCCGCCACCATGGACTACAAAGACCATGAC<br>GGTGATTATAAAGATCATGACATCGATTACAAGGATGACGATGACAAAATGACCGCCAAGGCCGTAGACAAAATCCC<br>AGTAACTCTCAGTGGTTTTGTGCACCAGCTGTCTGACAACATCTACCCGGTGGAGGACCTCGCCGCCACGTCGGTGA<br>CCATCTTTCCCAATGCCGAACTGGGAGGCCCCTTTGACCAGATGAACGGAGTGGCCGGAGATGGCATGATCAACATT<br>GACATGACTGGAGAGAAGAGGTCGTTGGATCTCCCATATCCCAGCAGCTTTGCTCCCGTCTCTGCACCTAGAAACCA<br>GACCTTCACTTACATGGGCAAGTTCTCCATTGACCCTCAGTACCCTGGTGCCAGCTGCTACCCAGAAGGCATAATCA<br>ATATTGTGAGTGCAGGCATCTTGCAAGGGGTCACTTCCCCAGCTTCAACCACAGCCTCATCCAGCGTCACCTCTGCC<br>TCCCCCAACCCACTGGCCACAGGACCCCTGGGTGTGTGCACCATGTCCCAGACCCAGCCTGACCTGGACCACCTGTA<br>CTCTCCGCCACCGCCTCCTCCTCCTTATTCTGGCTGTGCAGGAGACCTCTACCAGGACCCTTCTGCGTTCCTGTCAG<br>CAGCCACCACCTCCACCTCTTCCTCTCTGGCCTACCCACCACCTCCTTCCTATCCATCCCCCAAGCCAGCCACGGAC<br>CCAGGTCTCTTCCCAATGATCCCAGACTATCCTGGATTCTTTCCATCTCAGTGCCAGAGAGACCTACATGGTACAGC<br>T
	**Fragment 2:**<br>AGACTATCCTGGATTCTTTCCATCTCAGTGCCAGAGAGACCTACATGGTACAGCTGGCCCAGACCGTAAGCCCTTTC<br>CCTGCCCACTGGACACCCTGCGGGTGCCCCCTCCACTCACTCCACTCTCTACAATCCGTAACTTTACCCTGGGGGGC<br>CCCAGTGCTGGGGTGACCGGACCAGGGGCCAGTGGAGGCAGCGAGGGACCCCGGCTGCCTGGTAGCAGCTCAGCAGC<br>AGCAGCAGCCGCTGCCGCAGCTGCCTATAACCCACACCACCTGCCACTGCGGCCCATTCTGAGGCCTCGCAAGTACC<br>CCAACAGACCCAGCAAGACGCCGGTGCACGAGAGGCCCTACCCGTGCCCAGCAGAAGGCTGCGACCGGCGGTTCTCC<br>CGCTCTGACGAGCTGACACGGCACATCCGAATCCACACTGGGCATAAGCCCTTCCAGTGTCGGATCTGCATGCGCAA<br>CTTCAGCCGCAGTGACCACCTCACCACCCATATCCGCACCCACACCGGTGAGAAGCCCTTCGCCTGTGACTACTGTG<br>GCCGAAAGTTTGCCCGGAGTGATGAGAGGAAGCGCCACACCAAGATCCACCTGAGACAGAAAGAGCGGAAAAGCAGT<br>GCCCCCTCTGCATCGGTGCCAGCCCCCTCTACAGCCTCCTGCTCTGGGGGCGTGCAGCCTGGGGGTACCCTGTGCAG<br>CAGTAACAGCAGCAGTCTTGGCGGAGGGCCGCTCGCCCCTTGCTCCTCTCGGACCCGGACACCTTGATAATCTAGAG<br>GGCCCGCGGTTCGAAGGTAAGCCTATCCCTAACCCTCTCCTC
pEF6-3xFLAG-NAB2	TATAGGGAGACCCAAGCTGGCTAGGTAAGCTTGGTACCGAGCTCGGATCCGCCACCATGGACTACAAAGACCATGAC<br>GGTGATTATAAAGATCATGACATCGATTACAAGGATGACGATGACAAAATGCATAGGGCTCCCAGCCCGACTGCTGA<br>ACAGCCTCCCGGGGGAGGCGACAGTGCAAGAAGAACCTTGCAACCGAGATTGAAACCCAGTGCCCGCGCCATGGCCC<br>TGCCGCGGACTCTGGGAGAGTTGCAACTTTACCGCGTGCTGCAACGAGCCAATCTGTTGTCCTACTATGAAACGTTT<br>ATCCAGCAGGGTGGAGACGATGTTCAGCAGTTGTGCGAAGCAGGTGAGGAAGAATTTCTCGAGATTATGGCGCTTGT<br>AGGTATGGCAACGAAGCCGCTTCACGTGCGAAGGCTTCAGAAAGCATTGCGAGAATGGGCGACCAACCCGGGTTTGT<br>TTAGTCAGCCCGTACCTGCTGTACCTGTCTCTAGTATTCCCCTTTTCAAGATTAGCGAGACGGCTGGTACGCGCAAG<br>GGATCAATGAGTAATGGGCATGGCTCTCCCGGAGAGAAGGCAGGTAGCGCGCGGAGCTTTTCTCCAAAGTCCCCGTT<br>GGAACTTGGTGAAAAGTTGTCTCCACTTCCAGGTGGGCCGGGTGCCGGAGATCCAAGAATCTGGCCAGGACGCTCCA<br>CACCAGAAAGTGATGTCGGGGCGGGTGGCGAAGAGGAAGCCGGTTCACCACCATTCTCCCCTCCAGCTGGTGGAGGT<br>GTACCTGAAGGCACCGGAGCCGGCGGGCTGGCGGCAGGTGGAACCGGTGGTGGTCCCGATCGCTTGGAGCCTGAAAT<br>GGTCCGGATGGTTGTGGAATCTGTAGAACGGATCTTTCGATCTTTCCCAAGGGGTGATGCGGGGGAAGTCACTAGTT<br>TGTTGAAGCTGAACAAGAAACTCGCTAGATCTGTGGGCCATATATTCGAGATGGATGATAACGATTCTCAAAAGGAG<br>GAGGAAATCAGAAAATACTCTATAATTTACGGCCGATTCGACTCTAAGCGGCGGGAAGGAAAGCAGCTGTCATTGCA<br>TGAACTTACTATTAACGAAGCCGCCGCACAATTCTGCATGCGGGATAACACCTTGCTGCTGCGCAGAGTCGAACTCT<br>TCAGTTTGAGCCGGCAGGTCGCTCGAGAGTCAACCTACCTGTCCTCACTCAAAGGGTCCAGACTGCACCCAGAAGAA<br>CTCGGGGGGCCCCCACTCAAAAAGCTTAAGCAGGAGGTGGGGGAACAATCTCACCCCGAGATCCAGCAACCTCCTCC<br>CGGTCCAGAGAGCTATGTGCCCCCATATCGCCCGAGTCTTGAGGAGGATAGCGCCTCCCTCAGTGGTGAGTCCTTGG<br>ACGGACACCTTCAGGCAGTGGGGTCCTGTCCTCGACTTACTCCTCCGCCAGCAGACCTGCCACTGGCGCTGCCCGCT<br>CATGGCCTGTGGAGTAGACACATTCTCCAACAAACTCTTATGGATGAGGGATTGCGACTGGCTCGGCTTGTCTCCCA<br>CGACAGGGTGGGCCGGCTCTCACCGTGCGTACCAGCGAAACCCCCGCTGGCTGAATTTGAAGAAGGCCTCCTCGATA<br>GGTGCCCCGCGCCCGGCCCACACCCTGCGCTTGTTGAGGGTAGGCGATCCAGCGTAAAGGTTGAAGCAGAGGCCTCC<br>AGACAATGATCTAGAGGGCCCGCGGTTCGAAGGTAAGCCTATCCCTAACCCTCTCCTC
pEF6-3xFLAG-IRF4	ATTGCGGATCCGCCACCATGGACTACAAAGACCATGACGGTGATTATAAAGATCATGACATCGATTACAAGGATGAC<br>GATGACAAAATGAACCTGGAGGGCGGCGGCCGAGGCGGAGAGTTCGGCATGAGCGCGGTGAGCTGCGGCAACGGGAA<br>GCTCCGCCAGTGGCTGATCGACCAGATCGACAGCGGCAAGTACCCCGGGCTGGTGTGGGAGAACGAGGAGAAGAGCA<br>TCTTCCGCATCCCCTGGAAGCACGCGGGCAAGCAGGACTACAACCGCGAGGAGGACGCCGCGCTCTTCAAGGCTTGG<br>GCACTGTTTAAAGGAAAGTTCCGAGAAGGCATCGACAAGCCGGACCCTCCCACCTGGAAGACGCGCCTGCGGTGCGC<br>TTTGAACAAGAGCAATGACTTTGAGGAACTGGTTGAGCGGAGCCAGCTGGACATCTCAGACCCGTACAAAGTGTACA<br>GGATTGTTCCTGAGGGAGCCAAAAAAGGAGCCAAGCAGCTCACCCTGGAGGACCCGCAGATGTCCATGAGCCACCCC<br>TACACCATGACAACGCCTTACCCTTCGCTCCCAGCCCAGCAGGTTCACAACTACATGATGCCACCCCTCGACCGAAG<br>CTGGAGGGACTACGTCCCGGATCAGCCACACCCGGAAATCCCGTACCAATGTCCCATGACGTTTGGACCCCGCGGCC<br>ACCACTGGCAAGGCCCAGCTTGTGAAAATGGTTGCCAGGTGACAGGAACCTTTTATGCTTGTGCCCCACCTGAGTCC<br>CAGGCTCCCGGAGTCCCCACAGAGCCAAGCATAAGGTCTGCCGAAGCCTTGGCGTTCTCAGACTGCCGGCTGCACAT<br>CTGCCTGTACTACCGGGAAATCCTCGTGAAGGAGCTGACCACGTCCAGCCCCGAGGGCTGCCGGATCTCCCATGGAC<br>ATACGTATGACGCCAGCAACCTGGACCAGGTCCTGTTCCCCTACCCAGAGGACAATGGCCAGAGGAAAAACATTGAG<br>AAGCTGCTGAGCCACCTGGAGAGGGGCGTGGTCCTCTGGATGGCCCCCGACGGGCTCTATGCGAAAAGACTGTGCCA<br>GAGCAGGATCTACTGGGACGGGCCCCTGGCGCTGTGCAACGACCGGCCCAACAAACTGGAGAGAGACCAGACCTGCA<br>AGCTCTTTGACACACAGCAGTTCTTGTCAGAGCTGCAAGCGTTTGCTCACCACGGCCGCTCCCTGCCAAGATTCCAG<br>GTGACTCTATGCTTTGGAGAGGAGTTTCCAGACCCTCAGAGGCAAAGAAAGCTCATCACAGCTCACGTAGAACCTCT<br>GCTAGCCAGACAACTATATTATTTTGCTCAACAAAACAGTGGACATTTCCTGAGGGGCTACGATTTACCAGAACACA<br>TCAGCAATCCAGAAGATTACCACAGATCTATCCGCCATTCCTCTATTCAAGAATGATAATCTAGAGTCAT
pEF6-3xFLAG-PU.1	Sequence published in [1]

Supplementary Table 2
siRNAs sequences

siRNA	Strand	Sequence (5'--3')[1]
siCTRL	Sense	cuuAcGcuGAGuAcuucGAdTsdT
	Antisense	UCGAAGuACUcAGCGuAAGdTsdT
siTET2_1212	Sense	accucAGGGcAGAucAAuudTsdT
	Antisense	AAUUGAUCUGCCCUGAGGUdTsdT
siEGR2_1132 (a)	Sense	cucuAcAAuccGuaAcuuudTsdT
	Antisense	AAAGUuACGGAUUGuAGAGdTsdT
siEGR2_2665 (b)	Sense	guAAAuGGGuuGccuuAuudTsdT
	Antisense	AAuAAGGcAACCcAUUuACdTsdT
siIRF4_2931 (a)	Sense	caGGAuAuuuAcuauuAcudTsdT
	Antisense	AGuAAuAGuAAAuAUCCUGdTsdT
siIRF4_2384 (b)	Sense	uguccuAcAAucuaGuAAudTsdT
	Antisense	AUuACuAGAUUGuAGGAcAdTsdT

[1]Chemical modification pattern:
A, G, U, C: RNA Nucleotide
a, g, u, c: 2´-O-Methyl-Nucleotide
dT: desoxy-T residue
s: Phosphorothioate

Supplementary Table 3
EpiTYPER Primers

	Primer Sequence (5'--3')		
Amplicon	Forward	Reverse	Genomic location (GRCh38/hg38)
CCL13 (Fig. 2d)	AGGAAGAGAGTTTGTGGTTTG AATAGTTAGAAGGA	CAGTAATACGACTCACTATAGGGAGAAGGCT CAACAAACACAAAAACACTACAAAAA	chr17:34356259-34356559
RAB15 (Fig. 2d)	AGGAAGAGAGTGGTAATTAGG GTAGAAGGATAATGGTTAA	CAGTAATACGACTCACTATAGGGAGAAGGCT TCTCCTCCTAACACAAACACAAACTC	chr14:64965677-64965967
USP20 (Fig. 2d)	AGGAAGAGAGGGAATTTTGTT ATTTTTTAGGGTGG	CAGTAATACGACTCACTATAGGGAGAAGGCT AAAACCACCATCCTCTAACTCTC	chr9:129839252-129839471
PPCDC (Fig. S2e)	AGGAAGAGAGGGTATTTTGTG GTTGATTTTTTTGG	CAGTAATACGACTCACTATAGGGAGAAGGCT ACCAAACTAATATTAAACTCCTAACCTCAA	chr15:75051084-75051292
HCRTR1 (Fig. S2e)	AGGAAGAGAGTTTTGTAGATT AGTGAGTGAGTGGAAGG	CAGTAATACGACTCACTATAGGGAGAAGGCT CCAAAAAAAACAACCCCTAAAATTC	chr1:31625344-31625648
(Fig. S2e)	AGGAAGAGAGGGGTGATGTAG GGGTGAATTTTATT	CAGTAATACGACTCACTATAGGGAGAAGGCT AACAAACACCTACCCAAAAACCC	chr8:143399602-143400034
TMIGD3 (Fig. S2f)	AGGAAGAGAGTTTTGTTTGTT TATTAATTTTGGAGGTTTA	CAGTAATACGACTCACTATAGGGAGAAGGCT CCTAAAAAATAATTCAATAATTCCAACTTT	chr1:111491097-111491464
CABP4 (Fig. S2f)	AGGAAGAGAGTGTTTTAGAAG TTTAGGTAGATGATTAGGT	CAGTAATACGACTCACTATAGGGAGAAGGCT CTCCTTAAACTACCAACCAATAAATTAATA	chr11:67452163-67452604
(Fig. S2f)	AGGAAGAGAGGGGGGAGGGGTA GTTAATGTTTGAGT	CAGTAATACGACTCACTATAGGGAGAAGGCT AAAAAAACTAATTCTATAACTAATCCCAAA	chr11:61749586-61750085
MAN1C1 (Fig. S2f)	AGGAAGAGAGTTTTATTAGTA TAAAGGGGTTTGTTTT	CAGTAATACGACTCACTATAGGGAGAAGGCT TCCAAATAACCTAATAATCTAAAATTCTAA	chr1:25695856-25696293
(Fig. 5f)	AGGAAGAGAGTTTGTGGGTGT TTGGGTTTTTTTAT	CAGTAATACGACTCACTATAGGGAGAAGGCT ATTCCAAAATTTTATCCTCTTCAAATATCA	chr2:111239165-111239537
(Fig. 5g)	AGGAAGAGAGAGAAGTAAATA AATGTTGAGAATGTGTTAG	CAGTAATACGACTCACTATAGGGAGAAGGCT TCCAAAACTCCATACAATAAAAATAC	chr8:8949947-8950168
(Fig. 5h)	AGGAAGAGAGTGGGTTTTGGA ATTTTATTATTTGGG	CAGTAATACGACTCACTATAGGGAGAAGGCT AAACTCACATAAATCCATCTCCTCC	chr10:75070419-75070720
(Fig. 5i)	AGGAAGAGAGGGGTAGGATAG TGTATTGTAGTGGAAGT	CAGTAATACGACTCACTATAGGGAGAAGGCT CCTTAATAATCAAAAAATAACAACAAAAC	chr14:103889639-103889892
TBC1D1 (Fig. S5e)	AGGAAGAGAGGTTATAGGTAG GTTGAGGTTAGATTTGGAA	CAGTAATACGACTCACTATAGGGAGAAGGCT AAATCCCATCAAAATTTCAATAAATC	chr4:37954547-37954793
ITGAM (Fig. S5f)	AGGAAGAGAGATTTTTTAGTG GGTATTTTTATTGGGTATT	CAGTAATACGACTCACTATAGGGAGAAGGCT AAAAAAAACTACCTAAACTCCCTTATAC	chr16:31332112-31332381
ITPR2 (Fig. S5g)	AGGAAGAGAGTGAGTAAGGGG TAGGAAGGTTATTTT	CAGTAATACGACTCACTATAGGGAGAAGGCT AACTTTTAAAACCCAAAACTTTATACTTCA	chr12:26728155-26728400
(Fig. S5h)	AGGAAGAGAGTGTTAGGTTGT TTTTGTTTTTTGATAAGTT	CAGTAATACGACTCACTATAGGGAGAAGGCT AAATCCCCTACTTCCATTCACAATC	chr2:218005061-218005375
BAX (Fig. S6a)	AGGAAGAGAGTTGGGGGTTTT AGTTTATTTTTTTT	CAGTAATACGACTCACTATAGGGAGAAGGCT ACCTAAATCCAACTCTTTAATACCC	chr19:48961384-48961645
SLC25A47 (Fig. S6b)	AGGAAGAGAGTGGAGGGTTTG TTTGTGAGTTAGG	CAGTAATACGACTCACTATAGGGAGAAGGCT ACCCTATTTCCCCTCTAACTTCCCTATA	chr14:100325124-100325387
TGM2 (Fig. S6c)	AGGAAGAGAGTAGGAATTTTT ATTGTTGGGTGGAGT	CAGTAATACGACTCACTATAGGGAGAAGGCT TAAAATATAAATAAAATCCCCCACCCTTCT	chr20:38141132-38141388
DOCK2 (Fig. S6d)	AGGAAGAGAGTGGTGAGAAGA GGTTGTTTGTTGTT	CAGTAATACGACTCACTATAGGGAGAAGGCT AATTAATCCCCACAATCAAAATTCATTATA	chr5:169805716-169805911

Supplementary Table 4
5hmC capture-sequencing data generated in this study
(primary samples, accession IDs: EGAD00001006603, E-MTAB-9928)

Cell type	Sample	Donor	Total reads[1]	FRIP (%)[2]	Peaks[3]
MO	5hmC capture	donor A	25546492	50.74	113739
moDC, 18h	5hmC capture	donor A	20838968	52.31	135896
moDC, 7d	5hmC capture	donor A	22608535	55.34	146134
MO	input	donor A	21158002	–	–
moDC, 18h	input	donor A	27016412	–	–
MO	5hmC capture	donor B	48853424	46.54	90292
moDC, 18h	5hmC capture	donor B	23159298	46.11	49824
moDC, 7d	5hmC capture	donor B	21878582	50.84	136447
MO	input	donor B	30130040	–	–
moDC, 18h	input	donor B	20176298	–	–

[1]Unique reads after mapping to human reference genome GRCh38
[2]Fraction of reads in peaks (FRIP), determined by running HOMER's findPeaks program in ''histone'' mode using default parameters and the combined donor-matched background (input)
[3]Number of peaks (determined by HOMER's findPeaks program in ''factor'' mode with parameters "-tbp 1 -fdr 0.00001" and the combined donor-matched background (input)

Supplementary Table 5
ChIP-sequencing data generated in this study
(primary samples, accession IDs: EGAD00001006602, E-MTAB-9927)

Cell type	Sample	IP	Donor	Total reads[1]	FRIP (%)[2]	Peaks[3]
MO	freshly isolated	PU.1 ChIP	donor A	24524025	24.68	76074
moDC, 18h	18h IL4/GM-CSF	PU.1 ChIP	donor A	19710693	31.52	95470
moDC, 7d	7d IL4/GM-CSF	PU.1 ChIP	donor A	18562882	15.19	62848
moDC, 18h	18h IL4/GM-CSF	Input	donor A	27897594	–	–
MO	freshly isolated	PU.1 ChIP	donor B	23760716	20.90	63222
moDC, 18h	18h IL4/GM-CSF	PU.1 ChIP	donor B	31792250	29.13	116428
moDC, 3d	3d IL4/GM-CSF	PU.1 ChIP	donor B	36297448	20.79	94119
moDC, 7d	7d IL4/GM-CSF	PU.1 ChIP	donor B	28152341	29.82	120217
moDC, 18h	7d IL4/GM-CSF	Input	donor B	23278914	–	–
moDC, 3d	mock transfected	PU.1 ChIP	donor C	18020914	12.19	50923
moDC, 3d	FLAG-PU1 mRNA transfected	FLAG-ChIP	donor C	16818221	17.54	78335
moDC, 3d	mock transfected	FLAG-ChIP	donor C	17163313	–	–
moDC, 3d	mock transfected	PU.1 ChIP	donor D	18276534	28.11	88888
moDC, 3d	FLAG-PU1 mRNA transfected	FLAG-ChIP	donor D	20022986	12.86	61269
moDC, 3d	mock transfected	FLAG-ChIP	donor D	20482638	–	–
moDC, 4d	FLAG-EGR2 mRNA transfected	FLAG-ChIP	donor E	26586788	7.47	35631
moDC, 4d	FLAG-IRF4 mRNA transfected	FLAG-ChIP	donor E	30363269	4.15	23649
moDC, 4d	mock transfected	FLAG-ChIP	donor E	27110423	–	–

[1]Unique reads after mapping to human reference genome GRCh38
[2]Fraction of reads in peaks (FRIP), determined by running HOMER's findPeaks program in ‘‘factor’’ mode using default parameters and the matching background (input)
[3]Number of peaks (determined by HOMER's findPeaks program with parameters "-style factor -tbp 1 -fdr 0.000001" and the matching background (input)

Supplementary Table 6
ATAC-sequencing data generated in this study
(primary samples, accession IDs: EGAD00001006601, E-MTAB-9926)

Cell type	Sample	donor	Total read pairs[1]	FRIP (%)[2]	Peaks[3]
MO	freshly isolated	donor A	84340866	67.99	107464
MO	freshly isolated	donor B	50560551	9.37	37334
MO	freshly isolated	donor C	47672453	7.10	33182
moDC	7d IL4/GM-CSF	donor A	56211295	44.20	106392
moDC	7d IL4/GM-CSF	donor D	31249221	26.64	63793
moDC	7d IL4/GM-CSF	donor E	30279713	62.43	135146
moDC	7d IL4/GM-CSF	donor C	43647739	65.80	129246
moDC, 7d	mock transfected	donor F	44527793	37.72	107380
moDC, 7d	siIRF4.a transfected	donor F	45641296	44.93	102133
moDC, 7d	siCTRL transfected	donor F	39182782	41.27	100338
moDC, 7d	siIRF4.b transfected	donor F	45408998	39.99	102326
moDC, 7d	siCTRL transfected	donor G	56908984	40.28	100159
moDC, 7d	siTET2 transfected	donor G	52024954	56.30	123990
moDC, 7d	siTET2 transfected	donor F	37912370	22.03	63629
moDC, 7d	siCTRL transfected	donor H	36169121	45.03	101940
moDC, 7d	siTET2 transfected	donor H	47103019	64.81	131017
moDC, 4d	mock transfected	donor I	31932091	51.25	88342
moDC, 4d	siEGR2.b transfected	donor I	32898676	14.77	33549
moDC, 4d	siCTRL transfected	donor J	41349460	24.23	65089
moDC, 4d	siEGR2.b transfected	donor J	36303964	32.13	63830
MAC, 7d	mock transfected	donor K	26373907	47.58	118312
MAC, 7d	siCTRL transfected	donor K	36248496	39.26	114793
MAC, 7d	mock transfected	donor L	38239563	69.36	147122
MAC, 7d	siCTRL transfected	donor L	36038751	62.43	137881

[1]Unique read pairs after mapping to human reference genome GRCh38
[2]Fraction of reads in peaks (FRIP), determined by running HOMER’s findPeaks with parameters "-region -size 150"
[3]Number of peaks (determined by HOMER’s findPeaks program with parameters "-region -size 250 -L 0 -F 5 -minDist 350 -fdr 0.00001 -ntagThreshold 10"

Supplementary Table 7
RNA-sequencing data generated in this study
(primary samples, accession IDs: EGAD00001006604, E-MTAB-9929)

Cell type	Sample	Donor	RIN[1]	Total reads	Uniquely Mapped Reads (%)[2]
MO	freshly isolated	donor A	9.7	29843300	80.27
MO	freshly isolated	donor B	9.3	53456066	85.60
MO	freshly isolated	donor C	9.7	53849809	90.15
MO	freshly isolated	donor D	9.7	58503430	87.51
MAC, 7d	mock (electroporation control)	donor E	9.9	40479286	90.04
MAC, 7d	mock (electroporation control)	donor F	9.4	42061245	91.22
MAC, 7d	mock (electroporation control)	donor G	9.5	43159887	49.02
moDC, 7d	untransfected control	donor A	9.1	36683742	81.90
moDC, 7d	untransfected control	donor B	9.1	41980337	85.39
moDC, 7d	untransfected control	donor C	7.4	63087941	91.10
moDC, 7d	untransfected control	donor D	9.1	56461592	85.88
moDC, 7d	mock (electroporation control)	donor B	9.2	39468070	88.30
moDC, 7d	mock (electroporation control)	donor C	8.0	64269589	89.04
moDC, 7d	mock (electroporation control)	donor D	9.1	60379539	91.60
moDC, 7d	mock (electroporation control)	donor E	9.0	42986973	82.06
moDC, 7d	mock (electroporation control)	donor F	9.6	37261048	90.16
moDC, 7d	mock (electroporation control)	donor G	9.2	46745415	68.07
moDC, 7d	siCTRL transfected	donor A	9.1	33388475	85.31
moDC, 7d	siCTRL transfected	donor B	9.1	44102490	87.33
moDC, 7d	siCTRL transfected	donor C	8.8	50227965	89.83
moDC, 7d	siCTRL transfected	donor D	9.2	67212155	89.56
moDC, 7d	siEGR2.a transfected	donor A	8.6	29770618	87.30
moDC, 7d	siEGR2.a transfected	donor B	8.8	48142095	86.51
moDC, 7d	siEGR2.a transfected	donor C	8.7	44528953	92.09
moDC, 7d	siEGR2.a transfected	donor D	9.3	55868602	91.64
moDC, 7d	siEGR2.b transfected	donor A	8.8	36851394	85.88
moDC, 7d	siEGR2.b transfected	donor B	7.9	42329027	88.55
moDC, 7d	siEGR2.b transfected	donor C	8.8	43784117	91.74
moDC, 7d	siEGR2.b transfected	donor D	9.2	54757829	93.31
moDC, 7d	siIRF4.a transfected	donor A	9.4	38746171	88.58
moDC, 7d	siIRF4.a transfected	donor B	8.8	35650719	87.25
moDC, 7d	siIRF4.a transfected	donor C	8.5	57209689	91.65
moDC, 7d	siIRF4.a transfected	donor D	8.6	54117254	87.96
moDC, 7d	siIRF4.b transfected	donor A	9.2	37606950	85.59
moDC, 7d	siIRF4.b transfected	donor B	9.3	48410363	85.66
moDC, 7d	siIRF4.b transfected	donor C	8.6	58189847	91.58
moDC, 7d	siIRF4.b transfected	donor D	8.9	66757445	87.89
moDC, 7d	siTET2 transfected	donor B	9.1	42284779	87.52
moDC, 7d	siTET2 transfected	donor C	8.0	70927043	92.07
moDC, 7d	siTET2 transfected	donor D	9.2	57001936	89.73

[1]RNA-Integrity Index (RIN) as measured using the TapeStation (Agilent)
[2]percentage of unique sequences after mapping to GRCh38

Supplementary Table 8
Published WGBS-sequencing data used in this study

Cell type	Accession number	Data	CpGs	Reference
moDC	GSM1565940	methylation levels for each covered CpG (hg19) converted to bedGraph	24617968	[2]
moDC	GSM1565942	methylation levels for each covered CpG (hg19) converted to bedGraph	23551701	[2]
moDC	GSM1565944	methylation levels for each covered CpG (hg19) converted to bedGraph	24445760	[2]
moDC	GSM1565946	methylation levels for each covered CpG (hg19) converted to bedGraph	24479420	[2]
moDC	GSM1565948	methylation levels for each covered CpG (hg19) converted to bedGraph	24427703	[2]
moDC	GSM1565950	methylation levels for each covered CpG (hg19) converted to bedGraph	24560919	[2]
CD14+CD16– MO from venous blood	EGAX00001086967	methylation levels for each covered CpG (hg19), bigWig converted to bedGraph	22667869	http://dcc.blueprint-epigenome.eu/
CD14+CD16– MO from venous blood	EGAX00001086968	methylation levels for each covered CpG (hg19), bigWig converted to bedGraph	23768442	http://dcc.blueprint-epigenome.eu/
CD14+CD16– MO from venous blood	EGAX00001086970	methylation levels for each covered CpG (hg19), bigWig converted to bedGraph	23652501	http://dcc.blueprint-epigenome.eu/
CD14+CD16– MO from venous blood	EGAX00001097774	methylation levels for each covered CpG (hg19), bigWig converted to bedGraph	24145754	http://dcc.blueprint-epigenome.eu/
CD38- B cells	ERS214672	methylation levels for each covered CpG (hg38), bigWig converted to bedGraph	24601631	https://epigenomesportal.ca
CD38- B cells	ERS214675	methylation levels for each covered CpG (hg38), bigWig converted to bedGraph	24932472	https://epigenomesportal.ca
CD38- B cells	ERS222266	methylation levels for each covered CpG (hg38), bigWig converted to bedGraph	23878548	https://epigenomesportal.ca
CD38- B cells	ERS523625	methylation levels for each covered CpG (hg38), bigWig converted to bedGraph	23633379	https://epigenomesportal.ca
CD8+ T cells	ERS214674	methylation levels for each covered CpG (hg38), bigWig converted to bedGraph	25029123	https://epigenomesportal.ca
CD8+ T cells	ERS222241	methylation levels for each covered CpG (hg38), bigWig converted to bedGraph	24488031	https://epigenomesportal.ca
CD8+ T cells	ERS317233	methylation levels for each covered CpG (hg38), bigWig converted to bedGraph	25482590	https://epigenomesportal.ca
CD8+ T cells	ERS433791	methylation levels for each covered CpG (hg38), bigWig converted to bedGraph	22165393	https://epigenomesportal.ca
NK cells	ERS222243	methylation levels for each covered CpG (hg38), bigWig converted to bedGraph	24729720	https://epigenomesportal.ca
NK cells	ERS317235	methylation levels for each covered CpG (hg38), bigWig converted to bedGraph	25327131	https://epigenomesportal.ca
NK cells	ERS763560	methylation levels for each covered CpG (hg38), bigWig converted to bedGraph	26961816	https://epigenomesportal.ca
Neutrophils	ERS208313	methylation levels for each covered CpG (hg38), bigWig converted to bedGraph	26244136	https://epigenomesportal.ca
Neutrophils	ERS227748	methylation levels for each covered CpG (hg38), bigWig converted to bedGraph	24960155	https://epigenomesportal.ca
Neutrophils	ERS227749	methylation levels for each covered CpG (hg38), bigWig converted to bedGraph	26025313	https://epigenomesportal.ca
Neutrophils	ERS661057	methylation levels for each covered CpG (hg38), bigWig converted to bedGraph	25195761	https://epigenomesportal.ca
Neutrophils	ERS661059	methylation levels for each covered CpG (hg38), bigWig converted to bedGraph	23925309	https://epigenomesportal.ca
Neutrophils	ERS661060	methylation levels for each covered CpG (hg38), bigWig converted to bedGraph	24906321	https://epigenomesportal.ca
Hepatocytes	IHECRE00001879.2	methylation levels for each covered CpG (hg38), bigWig converted to bedGraph	21708615	https://epigenomesportal.ca
Hepatocytes	IHECRE00001876.2	methylation levels for each covered CpG (hg38), bigWig converted to bedGraph	22059276	https://epigenomesportal.ca
Hepatocytes	IHECRE00001878.2	methylation levels for each covered CpG (hg38), bigWig converted to bedGraph	21382628	https://epigenomesportal.ca
Hepatocytes	IHECRE00001877.2	methylation levels for each covered CpG (hg38), bigWig converted to bedGraph	22177806	https://epigenomesportal.ca
CD34+ Progenitor	ERS337604	methylation levels for each covered CpG (hg38), bigWig converted to bedGraph	25664889	https://epigenomesportal.ca
CD34+ Progenitor	ERS666926	methylation levels for each covered CpG (hg38), bigWig converted to bedGraph	25790225	https://epigenomesportal.ca

Supplementary Table 9
Published ChIP-sequencing data used in this study

Cell type	Sample	IP	Accession number	Total reads[1]	FRIP (%)[2]	Peaks[3]	Reference
MO	freshly isolated	CEBPB ChIP	SRR333633	5489459	4.45	13275	[3]
MAC	7d culture in 2% AB serum	CEBPB ChIP	SRR333649, SRR333650, SRR333651, SRR333652	14793649	14.01	67734	[3]
MO/MAC	7d IL4/GM-CSF	IgG control	SRR333634, SRR333635, SRR333636	13754210	–	–	[3]
Liver		CEBPA ChIP	ERR235748, ERR235729, ERR235723, ERR235766	37071639	12.76	72762	[4]
Liver		Input	ERR235788, ERR235759	30055419	–	–	[4]

[1]Unique reads after mapping to human reference genome GRCh38
[2]Fraction of reads in peaks (FRIP), determined by running HOMER's findPeaks program in ''factor'' mode using default parameters and the matching background (input)
[3]Number of peaks (determined by HOMER's findPeaks program in ''factor'' mode using default parameters and the matching background (input))

Supplementary Table 10
Published ATAC-sequencing data used in this study

Cell type	Accession number	Total read pairs	FRIP (%)	Peaks	Reference
MO	SRR2920543	63854968	33.86	63647	[5]
MO	SRR2920487	21181211	21.25	24324	[5]
MO	SRR2920475	9713700	29.68	36278	[5]
moDC	SRR1725732	204024037	41.00	285569	[2]

Supplementary References

1. Minderjahn, J. *et al.* Mechanisms governing the pioneering and redistribution capabilities of the non-classical pioneer PU.1. *Nature communications* **11,** 402; 10.1038/s41467-019-13960-2 (2020).

2. Pacis, A. *et al.* Bacterial infection remodels the DNA methylation landscape of human dendritic cells. *Genome research* **25,** 1801–1811; 10.1101/gr.192005.115 (2015).

3. Pham, T.-H. *et al.* Dynamic epigenetic enhancer signatures reveal key transcription factors associated with monocytic differentiation states. *Blood* **119,** e161-71; 10.1182/blood-2012-01-402453 (2012).

4. Ballester, B. *et al.* Multi-species, multi-transcription factor binding highlights conserved control of tissue-specific biological pathways. *eLife* **3,** e02626; 10.7554/eLife.02626 (2014).

5. Corces, M. R. *et al.* Lineage-specific and single-cell chromatin accessibility charts human hematopoiesis and leukemia evolution. *Nature genetics* **48,** 1193–1203; 10.1038/ng.3646 (2016).
