# Supplementary material for: The epigenetic pioneer EGR2 initiates DNA demethylation in differentiating monocytes at both stable and transient binding sites
Source: Nat Commun. 2021 Mar 10;12:1556. doi: 10.1038/s41467-021-21661-y (PMC7946903; doi:10.1038/s41467-021-21661-y)
Supplement: Supplementary file 3 — Reporting summary. [file 41467_2021_21661_MOESM3_ESM.pdf]

nature research

Corresponding author(s): Michael Rehli

Last updated by author(s): Feb 2, 2021

Reporting Summary

Nature Research wishes to improve the reproducibility of the work that we publish. This form provides structure for consistency and transparency in reporting. For further information on Nature Research policies, see Authors & Referees and the Editorial Policy Checklist .

Statistics

For all statistical analyses, confirm that the following items are present in the figure legend, table legend, main text, or Methods section.

n/a	Confirmed	
☐	☒	The exact sample size (*n*) for each experimental group/condition, given as a discrete number and unit of measurement
☐	☒	A statement on whether measurements were taken from distinct samples or whether the same sample was measured repeatedly
☐	☒	The statistical test(s) used AND whether they are one- or two-sided<br>*Only common tests should be described solely by name; describe more complex techniques in the Methods section.*
☒	☐	A description of all covariates tested
☐	☒	A description of any assumptions or corrections, such as tests of normality and adjustment for multiple comparisons
☐	☒	A full description of the statistical parameters including central tendency (e.g. means) or other basic estimates (e.g. regression coefficient) AND variation (e.g. standard deviation) or associated estimates of uncertainty (e.g. confidence intervals)
☐	☒	For null hypothesis testing, the test statistic (e.g. *F*, *t*, *r*) with confidence intervals, effect sizes, degrees of freedom and *P* value noted<br>*Give P values as exact values whenever suitable.*
☒	☐	For Bayesian analysis, information on the choice of priors and Markov chain Monte Carlo settings
☒	☐	For hierarchical and complex designs, identification of the appropriate level for tests and full reporting of outcomes
☒	☐	Estimates of effect sizes (e.g. Cohen's *d*, Pearson's *r*), indicating how they were calculated

Our web collection on statistics for biologists contains articles on many of the points above.

Software and code

Policy information about availability of computer code

Data collection	No software was used for data collection
Data analysis	Bedtools (v2.27.1); CrossMap (v0.2.7); EpiTyper (v1.2); Bowtie2 (v2.3.4); HOMER (v4.9); Integrative Genomics Viewer (IGV; v2.4.6); Metascape (v3.0); R main package (v3.4.3); R-package: beanplot (v1.2); R package: edgeR (v3.20.8); R-package: gplots (v3.0.1.1); R-package: ggplot2 (v3.1.0); R-package: igraph (v1.2.5); R-package: Rtsne (v0.15); R-package: venneuler (v1.1.0); R-package: plotrix (v3.7); R-package: cqn (v1.24.0); STAR (v2.5.3a); bigWigMerge (v2); bedGraphToBigWig (v4); Adobe Illustrator (v25.1)

For manuscripts utilizing custom algorithms or software that are central to the research but not yet described in published literature, software must be made available to editors/reviewers. We strongly encourage code deposition in a community repository (e.g. GitHub). See the Nature Research guidelines for submitting code & software for further information.

Data

Policy information about availability of data

All manuscripts must include a data availability statement. This statement should provide the following information, where applicable:

- Accession codes, unique identifiers, or web links for publicly available datasets
- A list of figures that have associated raw data
- A description of any restrictions on data availability

The EGA study accession number for the NGS raw data is EGAS00001004784 [https://www.ebi.ac.uk/ega/studies/EGAS00001004784]. Processed data files (bigwig tracks and peak files for ATAC, ChIP and 5mC-capture data and read count tables for RNA-seq data) are deposited with ArrayExpress (accession numbers: E-MTAB-9926, E-MTAB-9927, E-MTAB-9928, E-MTAB-9929) [https://www.ebi.ac.uk/arrayexpress/experiments/E-MTAB-9926/, etc.]. The source data underlying Figs 1c,d,f,g, 2a-e, 3a,b,d-g, 4a-f, 5a-c,e-j, 6a,c, 7b,c,f,g and Supplementary Figs 1a,b,d, 2a,c,d-f, 3a,c,d,f, 4a,c, 5a,c,e,g-k and 6a-d are provided as a Source Data file.

Field-specific reporting

Please select the one below that is the best fit for your research. If you are not sure, read the appropriate sections before making your selection.

☒ Life sciences ☐ Behavioural & social sciences ☐ Ecological, evolutionary & environmental sciences

For a reference copy of the document with all sections, see nature.com/documents/nr-reporting-summary-flat.pdf

Life sciences study design

All studies must disclose on these points even when the disclosure is negative.

Sample size	We did not perform any patient or in-vivo analysis as all data was generated using cell-culture standards. Since experiments were concordant we generally performed experiments in duplicate or triplicate. In particular, RNA-seq experiments were performed at least in triplicate, ATAC 5mC-capture and PU.1 ChIP experiments were performed with at least in duplicate. FLAG ChIPs after mRNA overexpression of EGR2 and IRF4 were performed once since they had low background and peaks contained the expected motif signature and correlated well with (but were much more sensitive compared to) ChIP-seq experiments with commercial antibodies that were not included in this study.
Data exclusions	All data are provided, and no data points were excluded.
Replication	RNA-seq experiments were performed at least in triplicate, ATAC 5mC-capture and PU.1 ChIP experiments were performed with at least in duplicate. FLAG ChIPs after mRNA overexpression of EGR2 and IRF4 were performed once since they had low background and peaks contained the expected motif signature and correlated well with (but were much more sensitive compared to) ChIP-seq experiments with commercial antibodies that were not included in this study. All replicate experiments were concordant.
Randomization	Blood cell donors for this study were randomly chosen.
Blinding	Since we did not perform any patient or in-vivo analysis, experiments were not blinded. Each experiment was designed with proper controls, and samples for comparison were collected and analyzed under the same conditions

Reporting for specific materials, systems and methods

We require information from authors about some types of materials, experimental systems and methods used in many studies. Here, indicate whether each material, system or method listed is relevant to your study. If you are not sure if a list item applies to your research, read the appropriate section before selecting a response.

Materials & experimental systems

n/a	Involved in the study	
☐	☒	Antibodies
☐	☒	Eukaryotic cell lines
☒	☐	Palaeontology
☒	☐	Animals and other organisms
☐	☒	Human research participants
☒	☐	Clinical data

Methods

n/a	Involved in the study	
☐	☒	ChIP-seq
☒	☐	Flow cytometry
☒	☐	MRI-based neuroimaging

Antibodies

Antibodies used	Rabbit polyclonal anti-TET2, Bethyl, Cat#A304-247A; Mouse monoclonal anti-TET2, clone hT2H 21F11, Merck, Cat#MABE462; Normal mouse IgG, Santa Cruz, Cat#sc-2025; Mouse monoclonal anti-FLAG M2, Sigma, Cat#F3165; Rabbit polyclonal anti-Actin, Sigma, Cat#A2066; Rabbit polyclonal anti-PU.1, Santa Cruz, Cat#sc-352X; Goat polyclonal anti-IRF-4, Santa Cruz, Cat#sc-6059X; Mouse monoclonal anti-Egr-2, Santa Cruz, Cat#sc-293195; Goat anti-rabbit immunoglobulins/HRP, Dako, Cat#P0448; m-IgGκ BP-HRP, Santa Cruz, Cat#sc-516102; Rabbit anti-goat immunoglobulins/HRP, Dako, Cat#P0449;
Validation	Anti-TET2 (Bethyl, Cat#A304-247A) has been validated by Bethyl (https://www.bethyl.com/product/A304-247A) and in house using immunoblotting (including TET2 wt and knock-down cells). anti-TET2 (Merck, Cat#MABE462) has been validated by Millipore/Merck (https://www.merckmillipore.com/DE/de/product/Anti-TET2-Antibody-clone-hT2H-21F11,MM_NF-MABE462?) for Western, ChIP and IP. Anti-Actin (Sigma, Cat#A2066) has been validated by Sigma (https://www.sigmaaldrich.com/catalog/product/sigma/a2066) and in house using immunoblotting. Anti-PU.1 (Santa Cruz, Cat#sc-352X) has been validated by Santa Cruz and in house using immunoblotting and has previously been used for ChIP experiments in numerous studies (e.g. see Minderjahn et al. 2020 Nat Communications). Anti-IRF-4 (Santa Cruz, Cat#sc-6059X) has been validated by Santa Cruz (https://www.scbt.com/p/irf-4-antibody-m-17) and in house using immunoblotting (including IRF4 wt and knock-down cells); this antibody has been used in numerous previous publications (as referenced on the SantaCruz website. Anti-Egr-2 (Santa Cruz, Cat#sc-293195) has been validated by Santa Cruz (https://www.scbt.com/p/egr-2-antibody-1g5) and in house using immunoblotting (including EGR2 wt and knock-down cells).

Eukaryotic cell lines

Policy information about cell lines

Cell line source(s)	THP-1, DSMZ, Cat#ACC16
Authentication	We are routinely checking cell line characteristic CNVs by low coverage whole genome sequencing.
Mycoplasma contamination	All cell lines tested negative for mycoplasma contamination.
Commonly misidentified lines (See ICLAC register)	No cell lines from the ICLAC register were used.

Human research participants

Policy information about studies involving human research participants

Population characteristics	Blood cells from male healthy donors between 18-40 in age.
Recruitment	Voluntary blood donations from healthy donors recruited for clinical research purposes. Recruitment is not initiated by the researcher and hence not self-biased. Donors are pseudonymised.
Ethics oversight	Primary blood cell types, monocytes and monocyte-derived cells were used throughout most of the experiments. Collection of blood cells from healthy donors was performed in compliance with the Helsinki Declaration. All donors signed an informed consent. The leukapheresis procedure and subsequent purification of hematopoietic cell types were approved by the ethical committee of the University of Regensburg (reference number 12-101-0260).

Note that full information on the approval of the study protocol must also be provided in the manuscript.

ChIP-seq

Data deposition

- [x] Confirm that both raw and final processed data have been deposited in a public database such as GEO.
- [x] Confirm that you have deposited or provided access to graph files (e.g. BED files) for the called peaks.

Data access links
May remain private before publication.

The EGA study accession number for the NGS raw data is EGAS00001004784 [https://www.ebi.ac.uk/ega/studies/EGAS00001004784]. Processed data files (bigwig tracks and peak files for ATAC, ChIP and 5mC-capture data and read count tables for RNA-seq data) are deposited with ArrayExpress (accession numbers: E-MTAB-9926, E-MTAB-9927, E-MTAB-9928, E-MTAB-9929) [https://www.ebi.ac.uk/arrayexpress/experiments/E-MTAB-9926/, etc.].

Files in database submission

EGAN00002795357, ChIP.MO.PU.1-ChIP.donorA.R1.fastq.gz, ChIP.PU1.MO.donorA.peaks.txt, ChIP.MO.PU.1-ChIP.donorA.bigwig
EGAN00002795333, ChIP.moDC18h.PU.1-ChIP.donorA.R1.fastq.gz, ChIP.PU1.MO.donorB.peaks.txt, ChIP.MO.PU.1-ChIP.donorB.bigwig
EGAN00002795408, ChIP.moDC.PU.1-ChIP.donorA.R1.fastq.gz
EGAN00002795409, ChIP.moDC18h.Input-.donorA.R1.fastq.gz, ChIP.PU1.moDC.donorA.peaks.txt, ChIP.moDC.PU.1-ChIP.donorA.bigwig,
EGAN00002795416, ChIP.MO.PU.1-ChIP.donorB.R1.fastq.gz, ChIP.PU1.moDC.donorB.peaks.txt, ChIP.moDC.PU.1-ChIP.donorB.bigwig
EGAN00002795417, ChIP.moDC18h.PU.1-ChIP.donorB.R1.fastq.gz
EGAN00002795412, ChIP.moDC3d.PU.1-ChIP.donorB.R1.fastq.gz, ChIP.PU1.moDC18h.donorA.peaks.txt, ChIP.moDC18h.PU.1-ChIP.donorA.bigwig
EGAN00002795411, ChIP.moDC.PU.1-ChIP.donorB.R1.fastq.gz, ChIP.PU1.moDC18h.donorB.peaks.txt, ChIP.moDC18h.PU.1-ChIP.donorB.bigwig
EGAN00002795406, ChIP.moDC.Input .donorB.R1.fastq.gz, ChIP.FLAG.PU1mRNA.moDC3d.donorC.peaks.txt, ChIP.moDC3d.FLAG-PU1mRNA.FLAG-ChIP.donorC.bigwig
EGAN00002795403, ChIP.moDC3d.mock.PU1-ChIP.donorC.R1.fastq.gz, ChIP.FLAG.PU1mRNA.moDC3d.donorD.peaks.txt, ChIP.moDC3d.FLAG-PU1mRNA.FLAG-ChIP.donorD.bigwig
EGAN00002795405, ChIP.moDC3d.FLAG-PU1mRNA.FLAG-ChIP.donorC.R1.fastq.gz, ChIP.moDC3d.mock.FLAG-ChIP.donorC.bigwig
EGAN00002795402, ChIP.moDC3d.mock.FLAG-ChIP.donorC.R1.fastq.gz, ChIP.moDC3d.mock.FLAG-ChIP.donorD.bigwig, ,
EGAN00002795407, ChIP.moDC3d.mock.PU1-ChIP.donorD.R1.fastq.gz, ChIP.PU1.moDC3d.donorC.peaks.txt, ChIP.moDC3d.mock.PU1-ChIP.donorC.bigwig
EGAN00002795404, ChIP.moDC3d.FLAG-PU1mRNA.FLAG-ChIP.donorD.R1.fastq.gz, ChIP.PU1.moDC3d.donorD.peaks.txt, ChIP.moDC3d.mock.PU1-ChIP.donorD.bigwig
EGAN00002795410, ChIP.moDC3d.mock.FLAG-ChIP.donorD.R1.fastq.gz, ChIP.PU1.moDC3d.donorB.peaks.txt, ChIP.moDC3d.PU.1-ChIP.donorB.bigwig
EGAN00002795401, ChIP.moDC4d.FLAG-EGR2mRNA.FLAG-ChIP.donorE.R1.fastq.gz, ChIP.FLAG.EGR2mRNA.moDC4d.donorE.peaks.txt, ChIP.moDC4d.FLAG-EGR2mRNA.FLAG-ChIP.donorE.bigwig

	EGAN00002795400, ChIP.moDC4d.FLAG-IRF4mRNA.FLAG-ChIP.donorE.R1.fastq.gz, ChIP.FLAG.IRF4mRNA.moDC4d.donorE.peaks.txt, ChIP.moDC4d.FLAG-IRF4mRNA.FLAG-ChIP.donorE.bigwig
Genome browser session (e.g. UCSC)	not applicable

Methodology

Replicates	PU.1 ChIPs were performed in independent duplicates and showed good agreement. FLAG ChIPs after mRNA overexpression of EGR2 and IRF4 were performed once since they had low background and peaks contained the expected motif signature and correlated well with (but were much more sensitive compared to) ChIP-seq experiments with commercial antibodies that were not included in this study.
Sequencing depth	EGAN00002795357, ChIP.MO.PU.1-ChIP.donorA.R1.fastq.gz, 24524025 reads, 50bp, SE<br>EGAN00002795333, ChIP.moDC18h.PU.1-ChIP.donorA.R1.fastq.gz, 19710693 reads, 50bp, SE<br>EGAN00002795408, ChIP.moDC.PU.1-ChIP.donorA.R1.fastq.gz, 18562882 reads, 50bp, SE<br>EGAN00002795409, ChIP.moDC18h.Input-.donorA.R1.fastq.gz, 27897594 reads, 50bp, SE<br>EGAN00002795416, ChIP.MO.PU.1-ChIP.donorB.R1.fastq.gz, 23760716 reads, 50bp, SE<br>EGAN00002795417, ChIP.moDC18h.PU.1-ChIP.donorB.R1.fastq.gz, 31792250 reads, 50bp, SE<br>EGAN00002795412, ChIP.moDC3d.PU.1-ChIP.donorB.R1.fastq.gz, 36297448 reads, 50bp, SE<br>EGAN00002795411, ChIP.moDC.PU.1-ChIP.donorB.R1.fastq.gz, 28152341 reads, 50bp, SE<br>EGAN00002795406, ChIP.moDC.Input .donorB.R1.fastq.gz, 23278914 reads, 50bp, SE<br>EGAN00002795403, ChIP.moDC3d.mock.PU1-ChIP.donorC.R1.fastq.gz, 18020914 reads, 50bp, SE<br>EGAN00002795405, ChIP.moDC3d.FLAG-PU1mRNA.FLAG-ChIP.donorC.R1.fastq.gz, 16818221 reads, 50bp, SE<br>EGAN00002795402, ChIP.moDC3d.mock.FLAG-ChIP.donorC.R1.fastq.gz, 17163313 reads, 50bp, SE<br>EGAN00002795407, ChIP.moDC3d.mock.PU1-ChIP.donorD.R1.fastq.gz, 18276534 reads, 50bp, SE<br>EGAN00002795404, ChIP.moDC3d.FLAG-PU1mRNA.FLAG-ChIP.donorD.R1.fastq.gz, 20022986 reads, 50bp, SE<br>EGAN00002795410, ChIP.moDC3d.mock.FLAG-ChIP.donorD.R1.fastq.gz, 20482638 reads, 50bp, SE<br>EGAN00002795401, ChIP.moDC4d.FLAG-EGR2mRNA.FLAG-ChIP.donorE.R1.fastq.gz, 26586788 reads, 50bp, SE<br>EGAN00002795400, ChIP.moDC4d.FLAG-IRF4mRNA.FLAG-ChIP.donorE.R1.fastq.gz, 30363269 reads, 50bp, SE<br>EGAN00002795399, ChIP.moDC4d.mock.FLAG-ChIP.donorE.R1.fastq.gz, 27110423 reads, 50bp, SE
Antibodies	Mouse monoclonal anti-FLAG M2, Sigma, Cat#F3165; Rabbit polyclonal anti-PU.1, Santa Cruz, Cat#sc-352X
Peak calling parameters	TF ChIP-seq peaks were called using HOMER's findPeaks program in ''factor'' mode with -fdr 0.00001 to identify focal peaks. Peak sets were filtered by subtracting blacklisted genomic regions and by filtering out regions with a mappability <0.8. See methods section "ChIP-seq Analysis" for references.
Data quality	For QC, the fraction of reads in peaks (FRIP, summarized in Supplementary Table 5 and for published data in Supplementary Table 10) was determined by running HOMER's (v4.9) findPeaks program in ''factor'' or "histone" mode using default parameters and the appropriate matching background data set (either ChIP input, genomic DNA or control ChIP).
Software	Reads (single-end) were aligned to the human genome (GRCh38/hg38) using bowtie2. Analysis of ChIP-seq data was mostly performed with the HOMER software (v4.9). For details see methods section "ChIP-seq Analysis"
